# Testing behaviour change with an artificial intelligence chatbot in a randomized controlled study

**DOI:** 10.1057/s41271-024-00500-6

**Published:** 2024-07-26

**Authors:** Simon T. van Baal, Suong T. T. Le, Farhad Fatehi, Antonio Verdejo-Garcia, Jakob Hohwy

**Affiliations:** 1 Monash Centre for Consciousness and Contemplative Science, Monash University, Melbourne, VIC Australia; 2 Department of Psychology, University of Warwick, Coventry, UK; 3 Faculty of Medicine, Nursing and Health Sciences, Monash University, Melbourne, VIC Australia; 4 School of Psychological Sciences, Turner Institute for Brain and Mental Health, Monash University, Melbourne, VIC Australia; 5 Monash University, Clayton, VIC 3800 Australia

**Keywords:** Chatbot, Human–computer interaction, Artificial intelligence, Behaviour change, Digital health

## Abstract

Chatbots can effect large-scale behaviour change because they are accessible through social media, flexible, scalable, and gather data automatically. Yet research on the feasibility and effectiveness of chatbot-administered behaviour change interventions is sparse. The effectiveness of established behaviour change interventions when implemented in chatbots is not guaranteed, given the unique human–machine interaction dynamics. We pilot-tested chatbot-based behaviour change through information provision and embedded animations. We evaluated whether the chatbot could increase understanding and intentions to adopt protective behaviours during the pandemic. Fifty-nine culturally and linguistically diverse participants received a compassion intervention, an exponential growth intervention, or no intervention. We measured participants’ COVID-19 testing intentions and measured their staying-home attitudes before and after their chatbot interaction. We found reduced uncertainty about protective behaviours. The exponential growth intervention increased participants’ testing intentions. This study provides preliminary evidence that chatbots can spark behaviour change, with applications in diverse and underrepresented groups.

**Supplementary Information:**

The online version contains supplementary material available at 10.1057/s41271-024-00500-6.

## Key message

- Chatbots can be equipped with animations to deliver standardised information and behaviour change interventions.
- We found preliminary evidence that using chatbots for behaviour change may be effective when users seek information independently.

**Supplementary Information:**

The online version contains supplementary material available at 10.1057/s41271-024-00500-6.

## Introduction

Chatbots are an increasingly popular, new human–computer interaction model, usually based on artificial intelligence conversational agents [1]. They have diverse applications in business, government, and research, including virtual personal assistants (such as Siri, Alexa, ChatGPT), digital health, government information provision, and customer support [1, 2].

Yet research on people’s responses to chatbots is in its infancy, even though chatbots could play an important role in judgment and decision making, marketing and consumer research, and behavioural economics. Chatbots may be used not just to inform, but to change behaviour in various ways: they can increase compliance with medical treatment [3], stimulate the adoption of public health guidelines [4], and steer purchasing decisions. It remains unclear, however, which specific elements of a chatbot are the most influential levers of behaviour change, much less how to design, implement and rigorously evaluate these interventions. Past findings of robust conventional behavioural interventions might not be as effective when administered through chatbots due to prevalent distrust of machines (e.g., [5]).

Chatbots are particularly suitable for behavioural interventions because they generate user data passively through user-initiated conversations. Those developing and implementing interventions can receive real-time information about the effectiveness of those interventions (through follow-up questions, attrition, information-seeking behaviour, purchases), which then provides information for iterative improvements.

While scalability can be complex under dynamic workloads, it is possible to rapidly scale chatbots when embedded on various social media channels simultaneously [6]. Such social media accessibility is especially useful for reaching young and diverse audiences as they rely less on traditional media [7, 8]. This increases the impact of behaviour change interventions and potentially improves inclusion of marginalised populations and diversity of samples in scientific experiments (depending on the domain).

We tested whether an in-house developed chatbot can effectively administer interventions to influence COVID-19 protective behaviors. We embedded the chatbot in a domain where they are already prevalent and useful: public health communication.

### Public health communication

The pandemic presents a recent prominent case where institutions used chatbots to address challenges that were difficult to address through traditional media outlets [9]. Governments and other organisations used chatbots to disseminate public health information in a more accessible, condensed format. This format allows users to get answers to their specific questions directly, rather than scanning a government web page or news article.

Chatbots are capable of reaching specific vulnerable demographics. Young people, migrants and refugees in nations around the world often perceive public health recommendations as unclear [10, 11]. It is more common for such marginalised groups to attain information on COVID-19 through social media. Use of public broadcasters is less impactful for culturally and linguistically diverse communities [12]. Risk communication through social media is, therefore, valuable for reaching younger and more diverse audiences [7, 8], who are at a higher risk of contracting and dying of COVID-19 [13, 14] due to significant health inequities and lower rates of vaccine uptake [15, 16]. It is possible to embed chhatbots on social media platforms, program them to reply in multiple languages, and accomodate low literacy rates [3].

Another dimension to public health communication, and risk communication involves public sentiment and the level of risk. If risk levels are high, but public awareness or outrage are low, (i.e., public relations; for an overview, see [17]), chatbots are unlikely to be useful to public health communication because chatbot interactions depend on user-initiation. When public outrage is high or moderate, chatbots can calm, provide factual messaging, tailored to a context. Complexity of public health crises inhibits traditional communication [18–20], but woth pre-training, chatbots can handle complex topics, prompting users only with aspects pertinent to the user’s situation.

**Fig. 1:**
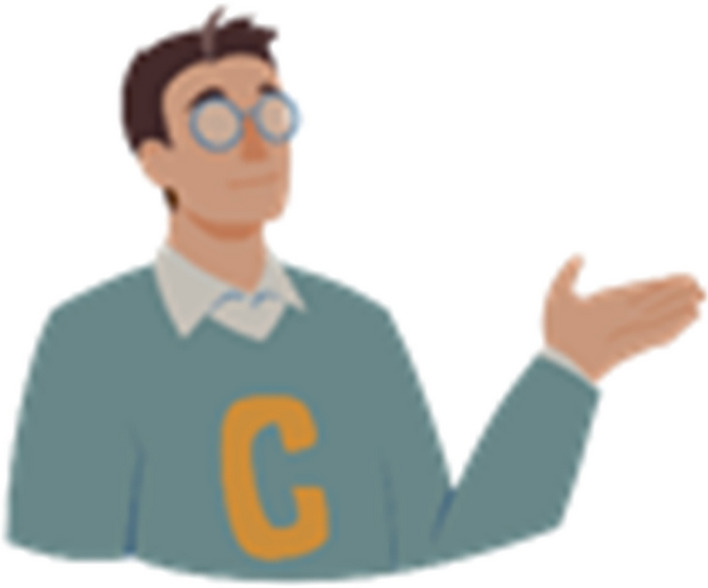
Cory COVID-Bot’s avatar is a friendly middle-aged librarian

To leverage the potential advantages of chatbots in reaching key demographics during the COVID-19 pandemic, we developed a chatbot and evaluated its ability to increase people’s propensity to test for COVID-19 if they experienced symptoms [21], and to reduce considerable discrepancies between people’s judgment about the acceptability of leaving the house for different reasons during a lockdown [22]. The chatbot, called Cory COVID-Bot (see Fig. 1), can converse in English and Vietnamese, and provides information on symptoms related to COVID-19, when to contact a medical professional, recommended protective behaviours, and factors relevant to spread of the virus. this chatbot has one major innovation: he can administer behavioural interventions through edutainment animation clips, deployed at appropriate points in conversations.

### Behaviour change interventions

Public health communication strategies may be augmented by behavioural interventions to stimulate change in people’s decision making [23, 24]. Thus, chatbots may be more effective at reaching public health policy goals if they effectively administer behavioural interventions.

To provide proof of concept for chatbot-based behavioural interventions for the key demographics identified earlier, we chose two messages that could be delivered through animations without dialogue. We chose to incorporate an animation to address the exponential growth bias, which can cause underestimation of the rapidity and dangers of COVID-19 spread. Alerting people to the exponential growth of COVID-19 cases can increase willingness to socially distance [25, 26], but it is unknown whether it can also increase the likelihood that people get tested for COVID-19 when they have symptoms. We incorporated an animation that elicits compassion for people affected by COVID-19. There is also evidence that eliciting compassion can increase the motivation for social distancing and wearing masks, as well as decrease urges to disobey public health guidelines [27, 28].

We set up automatic deployment of these interventions within Cory COVID-Bot when participants initiated queries about COVID-19 symptoms. We administered the interventions through animations to overcome barriers in age, cognition, health literacy, and English language fluency. We designed our chatbot to inform users about symptoms of COVID-19, protective behaviours, how the virus spreads, other relevant information pertaining to the disease–and explicitly to change their behaviour.

Thus, the aims of this study are also two-fold. First, we investigated whether the information provision is sufficient to increase understanding of appropriate behaviours related to COVID-19. Second, we investigated whether prompting compassion and alerting users to exponential growth in viral transmission can increase intentions to get tested. To test these aims, we test the effectiveness of the exponential growth and compassion-based animations for enhancing people’s perception of the importance of testing in the public health response, and for increasing users’ likelihood of getting tested. We then analysed our chatbot’s ability to remove people’s uncertainty about the acceptability of leaving their home for various reasons during a lockdown.

## Data and methods

### Study design

We conducted a pilot randomised controlled study using a single-blinded, between-group design, with two intervention groups and a control group. We recruited a total of 59 participants using random volunteer sampling through community advertisements.

We conducted the current experiment on 4 days in October 2020: 19, 21, 28 and 29, based on the Unified Theory of Acceptance and Use of Technology (UTUAT) framework [29]. To use chatbox as a public health tool, we applied the UTUAT framework to gauge behavioural intentions of using the chatbot after the testing had concluded. We assessed the main factors of UTUAT (performance expectancy, effort expectancy, social influence, and facilitating conditions) by asking participants about the likelihood of recommending the chatbot to their friends and by administering the Digital Behaviour Change Intervention Engagement Scale (DBCI; [30]), as described below.

We administered the alpha prototype of Cory COVID-Bot with manual switching between conditions, such that the three groups tested the chatbot sequentially (the control group, then the compassion group, then the exponential growth group). Because we completed all chatbot interactions on weekdays with slots during and after work hours, the risk of selection bias was limited.

Monash Health granted ethics approval under identifier HREC/69725/MonH-2020-237291(v3).

### Participants

The participants resided in Melbourne, in the Australian state of Victoria. We recruited them using random volunteer sampling through social media advertisements. They participated during Victoria’s second wave of COVID-19. At that time, it was clear from media reports and overall public sentiment that acceptance of an aggressive suppression strategy in Australia was fragile, as Victorians had experienced one of the strictest lockdowns in the world to date.

We selected participants to represent one of three target populations: 18–29 years of age, temporary visa holders, or Vietnamese nationals, among those who were fluent in English or Vietnamese. We compensated participants with an AUD 50 supermarket voucher upon completion of the exit survey after their interaction with Cory COVID-Bot.

Of the 59 participants, 11 participants forgot to fill out the pre-test survey, and 2 forgot to fill out the post-test survey, but all tested Cory COVID-Bot. Thus, 46 participants completed the entire study.

We randomly allocated participants to one of three groups: exponential growth, compassion, or control. After completion by each group, we manually shifted the software for the next.

### Assessment procedure

Participants used their own smartphone devices for testing Cory COVID-Bot, and we used the video conferencing programme Zoom for the duration of the supervised interaction with the chatbot.

The avatar for this chatbot is a knowledgeable, friendly middle-aged librarian (see Fig. 1). He uses emojis to seem more human-like which is associated with more effective conversations [3]. We designed the avatar to have an interactive and engaging interface, and to speak in simple English or Vietnamese given the potential low literacy rates of of users.

We elicited participants’ attitudes about staying home (as part of the public health orders) in 30 different scenarios adapted from Van Baal et al. [31]. We prompted participants with reasons for going out, such as “Someone wants to go for a walk in the park at 5 pm. It is a popular neighbourhood park with narrow footpaths near their house”, and asked “How certain are you that it is alright for them to leave the house?”. These scenarios fall in three different risk categories (minimal risk, low risk, high risk). Participants responded on a visual analogue scale ranging from “Completely certain it is not alright” to “Completely certain it is alright”, with the middle of the range representing uncertainty about the right course of action (for more details, see 31).

To elicit participants’ perceptions of the importance of testing, we had the librarian ask “How important do you think it is to get tested if you experience symptoms?”. They answered using a visual analogue scale ranging from “Not important at all” to “Extremely important”. The exponential growth and compassion behavioural animations showed how exponential growth of cases occurs and the painful separation of a family due to COVID-19, respectively. We then related these concepts to the importance of testing to limit disease transmission. The 20 and 51s animations relied on images and emotive music and contained no spoken or written words to maintain accessibility for people from different language groups.

Subsequent to the dialogue about symptoms (details on the procedure below)—and for two of the groups, animations—we elicited testing of participants’ intentions as follows: “How likely is it that you would get tested if you had symptoms?”, with the participants asked to respond with one of three options: “very likely”, “I don’t know”, or “very unlikely”.

We assessed participants’ acceptance of Cory COVID-Bot by administering the DCBI. The DBCI Engagement Scale posed questions about their experience with a behaviour change intervention in this format: “How much did you experience the following?” and eight response items: “Interest”, “Intrigue”, “Focus”, “Inattention”, “Distraction”, “Enjoyment”, “Pleasure”, “Annoyance”. Participants’ responses ranged from “not at all” (coded as 1), “moderately” [4], and “Extremely” [7]. The DBCI Engagement Scale includes questions about DBCI components with which the participant has interacted, and how much time the participant spent with the DBCI. We adjusted these questions slightly to fit with the context: “Which elements of Cory do you remember accessing without the experimenter?” and “How much time (in minutes) do you roughly think you spent interacting with Cory without the experimenter?”. We added to the DBCI Engagement Scale an item about the extent to which they experienced learning.

We assessed participants’ eligibility for the study through an initial survey collecting their informed consent, data on their demographic variables, and their availability for a video-conferencing session to test the chatbot. Subsequently, we scheduled a video-conferencing session by email, then asked each person to fill out a second survey (the pre-test survey) a day before their scheduled test and we sent a reminder. This survey provided a baseline for their perceived importance of testing in the response to COVID-19 and their attitudes on staying home or going out when faced with public health orders (detailed above).

The chatbot test consisted of two parts: a supervised part with an experimenter who guided each participant through a sequence of 18 questions to ask Cory COVID-Bot (in their own words; for the questions, see Supplementary Materials), and an unsupervised part for which the experimenter ended the zoom meeting. Then participants could ask Cory COVID-Bot whatever they wanted for 30 min. At the beginning of the test, each user received an SMS link to access Cory COVID-Bot through their device; the link directed them to a conversation with Cory COVID-Bot on Facebook Messenger.

Participants in one of the intervention groups would be shown an animation near the end of the structured section of the testing sequence. Then came the question about their testing intentions. The experimenter did not ask about their response. Participants chose whether to answer this question; 25 out of the 59 participants did so. We asked participants to fill out one more survey after they finished the unsupervised interaction with Cory COVID-Bot to qualify for payment. This survey included the DBCI Engagement Scale [30].

### Analysis

We used a cumulative link model with a logit link [32] to assess whether inclusion of the interventions mattered for the participants’ reports of whether they were likely to get tested for COVID-19 if they experienced symptoms. We chose this analysis method because it handles well ordinal data with multiple independent variables. The dependent variable had three levels: “very likely”, “I don’t know”, and “very unlikely”. The predictors included in the model were the group (exponential growth, compassion, or control), and the participants’ age and their sex as control variables.

We encountered convergence issues with the cumulative link model because the algorithm could not find uniquely determined parameters. As a result, we were unable to conduct pairwise post-hoc tests between groups based on the model, although we were able to analyse likelihood ratios of different models (such as testing whether the inclusion of each variable was important for the model). To conduct pairwise tests, we instead used one-sided Wilcoxon rank sum tests. We converted the factor levels to numerical values, where 1 signifies ‘very unlikely’, 2 signifies ‘I don’t know’, and 3 signifies ‘very likely’. To match the statistical test, we report medians with the Wilcoxon rank sum tests instead of means and standard deviations.

We also analysed whether participants reported higher perceived importance of getting tested after the interaction with Cory COVID-Bot with the manipulations versus without. For this analysis, we used a continuous ordinal regression [33, 34]. As noted, 11 participants forgot to fill out the pre-test survey, which caused partially overlapping data. Continuous ordinal regressions can handle non-normal, continuous, ordinal data, and are robust to partially overlapping data. The dependent variable for this model was the difference between participants’ perceived importance of getting tested after their interaction with the chatbot versus before. Responses were recorded on a visual analogue scale. We used condition, age, and sex as independent variables in this model.

To assess whether participants’ uncertainty about the acceptability of going out of their homes under the stay-at-home orders had decreased after the interaction with Cory COVID-Bot, we compared the absolute value on the VAS attitude scale before and after their interaction with Cory COVID-Bot. This procedure removed the valence (alright or not alright) but preserved the degree of certainty. For this analysis, we used continuous ordinal regression [34]. This model allowed us to estimate the effects of participants’ interaction with Cory COVID-Bot for each of the three risk levels in the original article [31]: high risk, low risk, and minimal risk. The independent variables in the model were the same as the above with the addition of the risk level of the considered scenario. P-values reported were considered significant at a false discovery rate corrected alpha of 0.05. For all analyses, we conducted programming in R [35].

## Results

There were 18 males in the sample with a mean age of 24.8 (*SD* = 3.4), 40 females with mean age of 25.9 (*SD* = 5.3), and one participant who did not provide their age or sex (Table 1). The different response rates led to varying sample composition over the tests we report in this paper.

**Table 1:** Sample demographics by condition

Female/Male	Attitudes & Perceived Testing Importance						Testing Likelihood					
	Compassion		Exponential		Control		Compassion		Exponential		Control	
	7/6		8/6		17/3		7/2		4/3		6/2	
	M (n)	SD	M (n)	SD	M (n)	SD	M (n)	SD	M (n)	SD	M (n)	SD
Australia	24.1 [11]	2.7	23.4 [8]	4.4	23.2 [9]	3.3	24.2 [8]	2.8	22.2 [6]	4.6	21.4 [5]	3.3
Other Migrants	23 [2]	2.8	28 [3]	5.6	26.6 [5]	0.9	21 [1]	-	33 [1]	-	26 [2]	0
Viet Nam	–	–	32.8 [9]	4.8	–	–	–	–	33 [1]	–	–	–

**Fig. 2:**
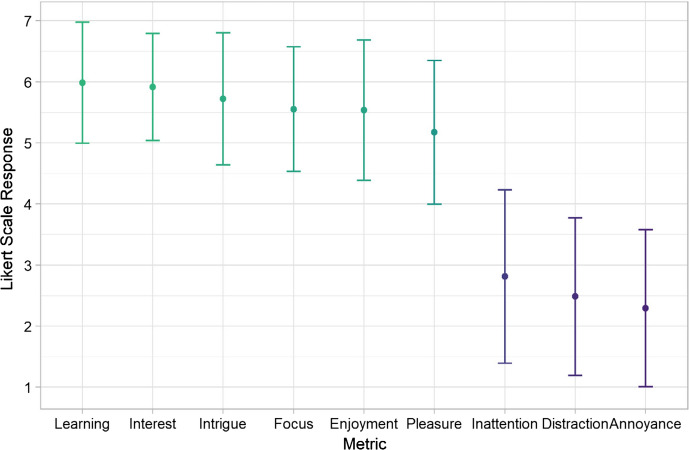
Participants’ responses on the DBCI Engagement Scale. All questions were coded between 1 and 7, error bars are standard deviations

The post-test evaluation was overwhelmingly positive. When asked how likely they were to recommend Cory COVID-Bot COVID-Bot to a friend, the average response was 8.9/10. Additionally, users indicated an average of 6 on a 1–7 Likert scale on whether they experienced learning. Figure 2 offers a depiction of participants’ responses on the DBCI Engagement Scale and our question on their experience of learning.

### The importance and likelihood of getting tested

**Fig.3:**
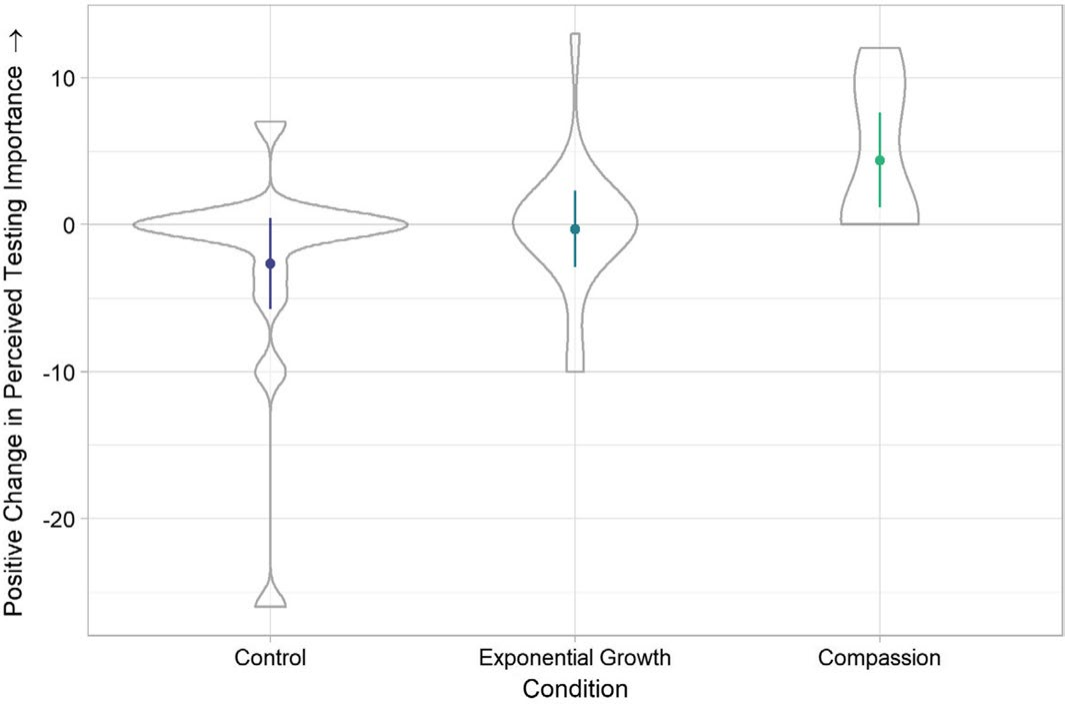
The difference between the perceived importance of testing after versus before the interaction with Cory COVID-Bot (y-axis), in the compassion, exponential growth, and control conditions (x-axis). Error bars are 95% CIs, violin plots represent datapoints

The experimental condition (Compassion, EFT, Control) significantly predicted participants’ perceptions of the importance of testing for COVID-19,* χ*^*2*^(2) = 15.978, *p* < 0.001. Participants reported a larger increase in perceived importance of getting tested for COVID-19 in the compassion condition (*M* = 4.38, *SD* = 4.91) than in the control condition (*M* = − 2.64, *SD* = 7.65), *b* = − 2.381, 95% CI [− 3.823, − 0.938], *t*(2) = 3.236, *p* = 0.01. We found no evidence for such an effect of the exponential growth manipulation (*M* = − 0.30, *SD* = 4.64), *b* = 0.056, 95% CI [− 1.171, 1.283], *t*(12) = 0.090, *p* = 0.92. Figure 3 shows for the difference in perceived importance of testing before the interaction with Cory COVID-Bot, and after the interaction with Cory COVID-Bot over the three conditions.

**Fig. 4:**
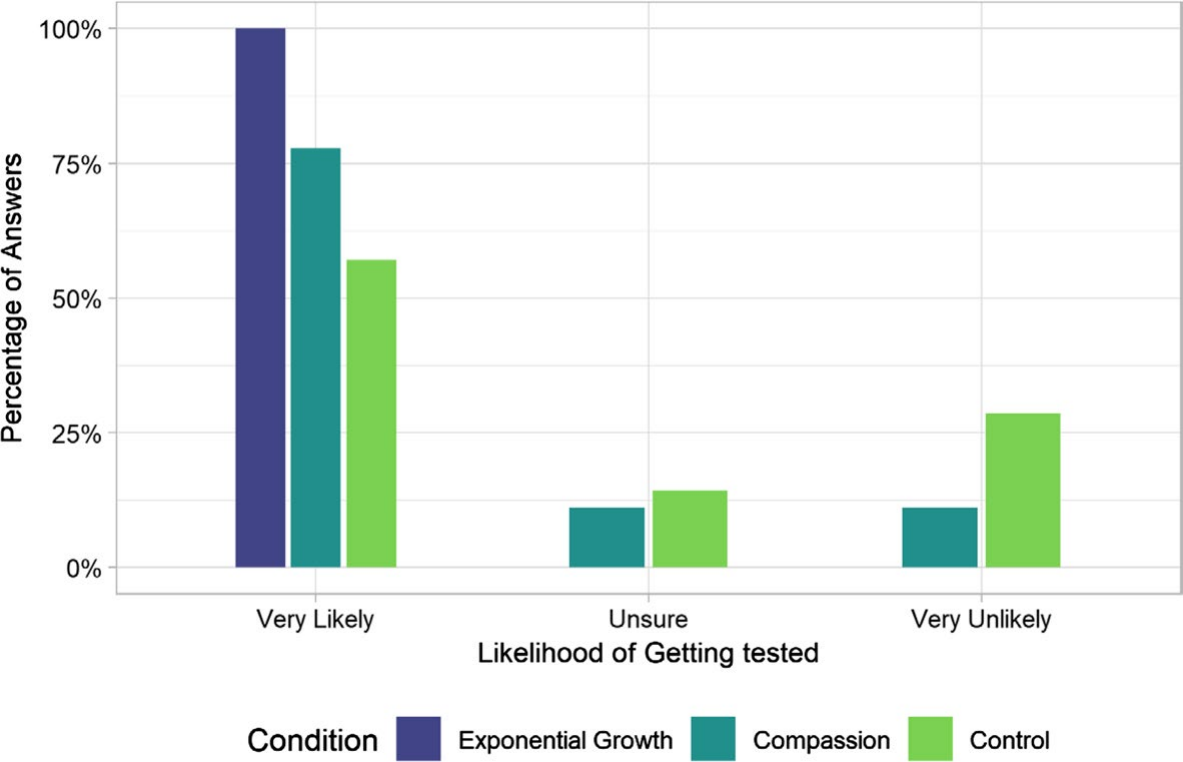
Participants reported likelihood of getting tested if they were to experience symptoms. The bars represent the percentage of the total answers in that group

The cumulative link model showed that including the group factor significantly improved the model for predicting people’s reported likelihood of getting tested *χ*^*2*^(2) = 6.146, *p* = 0.042. The Wilcoxon rank sum tests showed that participants indicated a higher likelihood of getting tested if the question was preceded by the exponential growth manipulation (*Mdn* = 3), compared to the control condition (*Mdn* = 2.29) although this difference was not significant after corrections for multiple comparisons, *z* = 1.989, *p* = 0.056. We also found no evidence that there was a difference between the reported likelihood to get tested in the compassion condition (*Mdn* = 2.59) versus the control condition, *z* = 1.034, *p* = 0.20. See Fig. 4 for a depiction of the frequencies of endorsing testing likelihoods across conditions.

### Attitudes toward the acceptability of leaving home

Likelihood ratio tests show that the inclusion of the timepoint variable (pre v post chatbot interaction), *χ*^*2*^(0.84) = 21.733, *p* < 0.0001, and the risk level of different scenarios, *χ*^*2*^(2.11) = 24.558, *p* < 0.0001, significantly explained participants’ certainty about leaving the house during a lockdown. The interaction between the timepoint and risk level variables was also significant, *χ*^*2*^(2.09) = 36.255, *p* < 0.0001.

There was a significant difference between certainty post-test (*M* = 32.45, *SD* = 16.00) versus pre-test (*M* = 28.76, *SD* = 16.06), *b* = − 0.665, 95% CI [− 0.865, − 0.466], *t*(74) = 6.548, *p* < 0.0001.

**Fig. 5:**
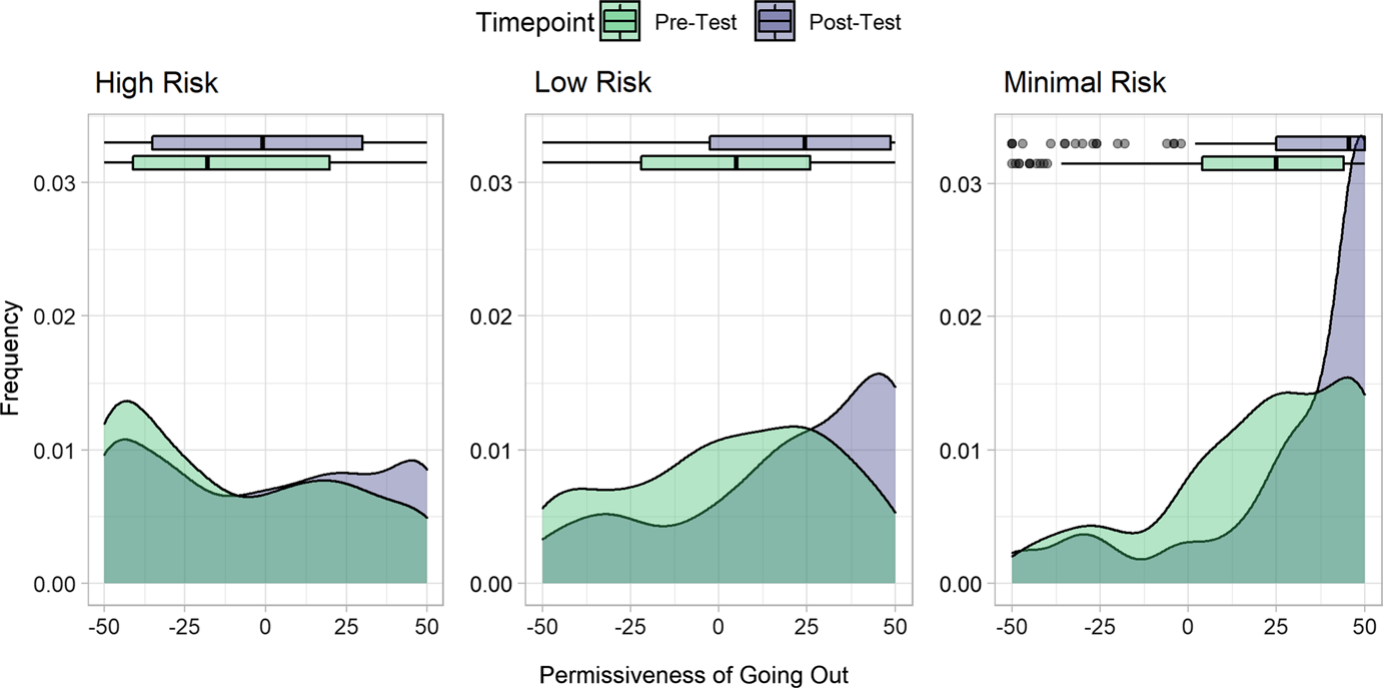
Certainty on whether it is alright to go out in various risk contexts; -50 represents “Completely certain it’s not alright to go out”, and 50 represents “Completely certain it is alright to go out”. The green area shows the density of responses before the chatbot test, while the blue area represents the responses after the chatbot test

The pairwise interaction coefficients show that participants’ certainty about the acceptability of going out for scenarios in the minimal and low risk categories was significantly higher in the post-test (*M* = 39.01, *SD* = 13.94; *M* = 32.30, *SD* = 15.89) than in the pre-test (*M* = 29.20, *SD* = 16.10; *M* = 24.85, *SD* = 15.53), respectively: *b* = − 1.206, − 0.818; 95% CI [− 1.587, − 0.825; − 1.190, − 0.476], *t*(74) = 6.204, 4.574; *ps* < 0.0001. There was no evidence, however that participants were more certain in the high risk scenarios post-test (*M* = 31.19, *SD* = 16.12) than pre-test (*M* = 31.02, *SD* = 15.96), *b* = 0.043, 95% CI [− 0.210, 0.295], *t*(74) = 0.332,* p* = 0.74 (Fig. 5).

## Discussion

Our study investigated the ability of an AI chatbot to administer COVID-19 protective behavioural interventions. A novel aspect of the study included assessment of whether effective behavioural interventions could be embedded in the natural cadence of a conversation with the user through animations without dialogue. We tested this in the context of the COVID-19 pandemic, where we aimed to enhance people’s understanding of the public health guidelines and increase the likelihood they would get tested for COVID-19 if they experienced symptoms.

The results show promising signs of the potential efficacy and scalability of using a chatbot to diminish ambiguity about guidelines and appropriate behaviour during an outbreak, as well as for administering behavioural interventions through animations, especially among diverse and marginalised populations. Participants indicated that their interaction with Cory COVID-Bot was useful and enjoyable. The behavioural interventions demonstrated signs of efficacy; the compassion intervention increased the perceived importance of testing, while participants in the exponential growth condition were much more likely to indicate they would get tested if they were to experience COVID-19 symptoms compared to those who did not receive this intervention. Participants also indicated much less uncertainty about whether it would be alright to leave their house during a lockdown.

The compassion intervention affected the perceived importance of testing more than thirty minutes after the chatbot test was completed. The animations we used to invoke compassion in people included no spoken or written language. This shows that those behavioural interventions, which are easy to understand for culturally and linguistically diverse people and others experiencing a language barrier, can be developed and embedded in chatbots. This result also extends findings that invoking compassion can promote social distancing [27], by showing that compassion may be used to stimulate the importance of compliance with other public health measures such as testing. The observation that compassion may be susceptible to short-term interventions accords well with the prosocial behaviour literature [36–38]. We found no evidence, however, that the compassion intervention increased people’s likelihood of getting tested if they experienced symptoms. This could be because the intervention did not overtly link the importance of testing to the behavioural outcome.

We detected that an animation alerting people to exponential growth increased participants’ reported likelihood of getting tested should they experience COVID-19 symptoms. This is an interesting finding as the initial results on exponential growth bias have recently come under scrutiny [39]. Our study finds that alerting people to exponential growth can enhance compliance with public health measures in a pandemic [25, 26], suggesting that exponential growth bias may be playing a role in people’s risk perception.

Participants also evolved from a state of ambiguity when asked about their judgments on whether they were certain that it was alright to leave home in predetermined scenarios [31], to more definitive responses after interacting with Cory COVID-Bot (see Fig. 3). Situations where people experience ambiguity concerning the restrictions and guidelines of the government, and the permissibility of their actions, are the situations that Cory COVID-Bot is intended to resolve by creating clarity for the user. Emerging evidence also shows that the many uncertainties associated with the pandemic caused mental health issues, especially for those with an intolerance for uncertainty [40–42]. Participants were also likely to feel less impeded for doing things that were allowed but e shrouded in uncertainty before their interaction with Cory COVID-Bot, leading to better decision making, especially in scenarios with low risk.

The results, thus, provide initial evidence that behaviour change interventions may be effective when incorporated into chatbot conversations at appropriate times. It is important to keep in mind when these interventions will be most effective. Chatbot-based interventions are more likely to be effective when users perceive some degree of risk because it engenders attentiveness and likelihood of engaging in conversation [17]. Chatbots have already shown to be good at answering medical questions, and are continually improving [43]. It is likely that chatbots can play an important role in future public health crises, and approaches such as ours should be thoroughly tested for effectiveness.

## Limitations

The current study aimed to provide proof of concept for the effective administration of interventions leveraging the capabilities and advantages of a chatbot. The findings here should not be taken as a rigorous test of which interventions do and do not work during a pandemic, but they might nonetheless provide a starting point for future research.

Our sample size was small and there are various ways to improve the effectiveness of the design and the data collection capabilities of chatbots. Our design encouraged participants to answer any questions Cory COVID-Bot asked them (aiming at the question of whether they would be likely to get tested for COVID-19), but fewer than half of the participants answered Cory COVID-Bot when asked the target question. A better option would be to increase the sample size and link the chatbot to a website for purchasing COVID-19 home testing kits. This type of design would provide evidence for behaviour change, which is a more direct measure than relying on reported intentions.

Our testing framework was labour intensive—it is important to get direct user feedback, especially when adopting the UTUAT framework, in the early stages of the chatbot implementation process. Once this phase is completed and the chatbot is rolled-out, however, the marginal costs of testing new users will be minimal.

A potential limitation for chatbot research is that the social media platforms on which chatbots are often deployed provide limited or no demographic information on their users; this could hamper moderator analysis. Such issues could be resolved by releasing surveys with incentives through the chatbot, to link to participants’ responses.

## Conclusions

We have shown that chatbots can reduce uncertainty for their users, but also that they can be equipped with behaviour change interventions in the form of animations. Relying mainly on visual cues could help suppress costs for researchers, health practitioners, and public officials who wish to reach young people or culturally and linguistically diverse audiences, or both, while maintaining the effectiveness of the interventions.

### Supplementary Information

Below is the link to the electronic supplementary material.

Supplementary file1 (DOCX 15 KB)

## Data Availability

The animations, data and code are publicly available [44, 45].
