# Supplementary material for: Testing behaviour change with an artificial intelligence chatbot in a randomized controlled study
Source: J Public Health Policy. 2024 Jul 26;45(3):506–22. doi: 10.1057/s41271-024-00500-6 (PMC11315670; doi:10.1057/s41271-024-00500-6)
Supplement: Supplementary file 1 — Supplementary file1 (DOCX 15 KB) [file 41271_2024_500_MOESM1_ESM.docx]

Supplementary Materials

# **Cory Chatbot Questions**

Participants asked Cory COVID-Bot questions in a structured fashion, asking all of the below in their own words, in order. Afterwards, participants had 30 minutes to do further free chatting with the chatbot, before they were supposed to complete the post-test survey.

1. Are outdoor team sports allowed? If not, when?
2. Can I go to the hairdresser?
3. Can I go to the park?
4. Can I go to a skate park?
5. Can I hang out with 10 friends outside?
6. Is Social Distancing still important?
7. For what reasons can I leave my house?
8. I'm lonely, what can I do about it?
9. How should I take care of my mental health?
10. Can I visit my friends' house?
11. Can I get financial support?
12. Is there help for tough break ups?
13. I know someone that suffers from abuse, what can I do?
14. What symptoms are related to COVID-19?
15. What should you do when you have symptoms?
16. How and where do I get tested?
17. Someone got tested at work, should I get tested?
18. A friend tested positive, should I get tested?
